# Supplementary figures and images for: Modelling smallholder farmers’ preferences for soil fertility management technologies in Benin: A stated preference approach
Source: PLoS One. 2021 Jun 30;16(6):e0253412. doi: 10.1371/journal.pone.0253412 (PMC8244892; doi:10.1371/journal.pone.0253412)

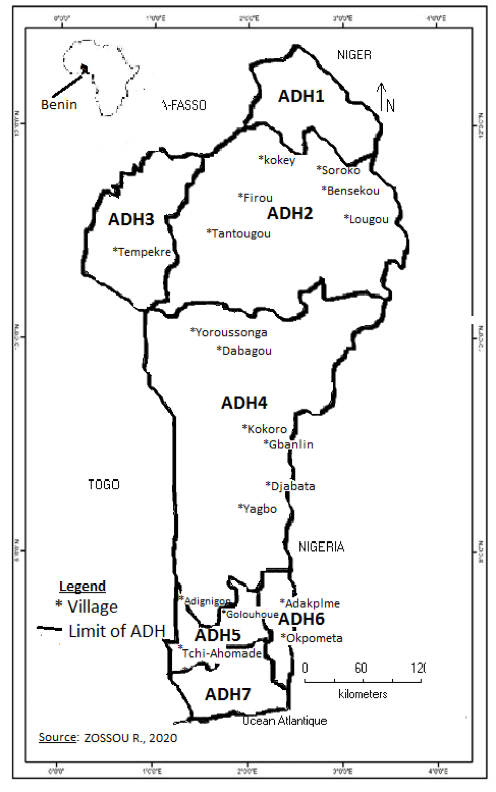


**Fig 1. Study zone**

Supplement: S1 Fig — (DOCX) [file pone.0253412.s001.docx]
