# Supplementary material for: Modelling smallholder farmers’ preferences for soil fertility management technologies in Benin: A stated preference approach
Source: PLoS One. 2021 Jun 30;16(6):e0253412. doi: 10.1371/journal.pone.0253412 (PMC8244892; doi:10.1371/journal.pone.0253412)
Supplement: S2 Fig — (DOCX) [file pone.0253412.s002.docx]

| Choice set 2 | | | | | | |
| --- | --- | --- | --- | --- | --- | --- |
|  | | Practice **#5** | Practice **#6**  **herbaceous**  **legumes**  **(*Mucuna,***  ***Ashynomenae,***  ***Stylosanthes*)** | Practical **#7**  **Microorganism**  ***(eg.***  ***mushrooms***) | Practice **#8**  **Crop rotation** | None of these practices interest me, i prefer to maintain current practice  (*Option q0)* |
| **Restoration time** | | Long | Short | Long | Short |  |
| **Accessibility** | | **Easy** | Difficult | Difficult | **Easy** |  |
| **Possibility of obtaining edible by-products** | | Impossible | Impossible | Possible | Possible |  |
| **Soil fertility**  **retention time** | | Temporary (*1 production*  *campaign*) | Temporary  (*1*  *production*  *campaign*) | Long  (*more than one*  *production campaign*) | Long  (*more than one*  *production campaign*) |  |
| **Regular control (**frequency of maintenance of the plot) | | Regular control | Less  control | Regular  control | Less  control |  |
| **Purchase cost**  **(CFAF per hectare)** | | **70 000** | **70 000** | **100 000** | **70 000** |  |
| ***Which of these***  ***practices do***  ***you choose?*** | ***Plot1*** |  |  |  |  |  |
|  | ***Plot2*** |  |  |  |  |  |
|  | ***Plot3*** |  |  |  |  |  |

**Fig 2. Example of a set of cards proposed during the interview**
