# Supplementary material for: Modelling smallholder farmers’ preferences for soil fertility management technologies in Benin: A stated preference approach
Source: PLoS One. 2021 Jun 30;16(6):e0253412. doi: 10.1371/journal.pone.0253412 (PMC8244892; doi:10.1371/journal.pone.0253412)
Supplement: S3 Fig — (DOCX) [file pone.0253412.s003.docx]

Degree I (Without limitations); Degree II (Weak limitations); Degree III (Average limitations); Degree IV (Severe Limitations);

Degree IV (Very severe limitations)

**Fig 3. Degree of intensity of associated limitations, according to ADHs**
