# Supplementary material for: Modelling smallholder farmers’ preferences for soil fertility management technologies in Benin: A stated preference approach
Source: PLoS One. 2021 Jun 30;16(6):e0253412. doi: 10.1371/journal.pone.0253412 (PMC8244892; doi:10.1371/journal.pone.0253412)
Supplement: S4 Fig — (DOCX) [file pone.0253412.s004.docx]

**Fig 4. Estimate of the willingness-to-adopt from the three latent classes model**
