# Supplementary material for: Modelling smallholder farmers’ preferences for soil fertility management technologies in Benin: A stated preference approach
Source: PLoS One. 2021 Jun 30;16(6):e0253412. doi: 10.1371/journal.pone.0253412 (PMC8244892; doi:10.1371/journal.pone.0253412)
Supplement: S1 Table — (DOCX) [file pone.0253412.s005.docx]

**Table 1. Criteria for assessing the degrees of limitation of soil chemical parameters**

| **Soil chemical parameters** | **Degrees of limitation** | | | | |
| --- | --- | --- | --- | --- | --- |
|  | **Degree I**  **(Without limitations)** | **Degree II (Weak limitations)** | **Degree II (Weak limitations)** | **Degree: IV (Severe Limitations)** | **Degree: V (Very severe limitations)** |
| **Organic matter** | > 2 | 2 - 1.5 | 1.5 – 1 | 1 - 0.5 | < 0.5 |
| **Total nitrogen** | > 0.08 | 0.08 - 0.06 | 0.06 - 0.045 | 0.045 - 0.03 | < 0.03 |
| **P ppm (Bray _1_)** | > 20 | 20 - 15 | 15 - 10 | 10 - 5 | < 5 |
| **K (meq/100 g of soil)** | > 0.4 | 0.4 - 0.3 | 0.3 - 0.2 | 0.2 - 0.1 | < 0.1 |
| **Sum of exchangeable bases (meq/100 g of soil)** | > 10 | 10 - 7.5 | 7.5 - 5 | 5 - 2 | < 2 |
| **Base Saturation (V)** | > 60 | 60 - 50 | 50 - 30 | 30 - 15 | < 15 |
| **CEC (meq/100 g of soil)** | > 25 | 25 - 15 | 15 - 10 | 10 - 5 | < 5 |
| **pH** | 6.5 - 6.0 | 6.0 - 5.5 | 5.5 - 5.3 | 5.3 - 5.2 | < 5.2 |
|  | 6.5 - 7.8 | 6.5 - 7.8 | 7.8 - 8.3 | 8.3 - 8.5 | > 8.5 |
