# Supplementary material for: Modelling smallholder farmers’ preferences for soil fertility management technologies in Benin: A stated preference approach
Source: PLoS One. 2021 Jun 30;16(6):e0253412. doi: 10.1371/journal.pone.0253412 (PMC8244892; doi:10.1371/journal.pone.0253412)
Supplement: S2 Table — (DOCX) [file pone.0253412.s006.docx]

**Table 2. Attributes and associated attribute levels**

| **Attributes** | **Attributes levels** |
| --- | --- |
| Restoration time | 1 = Short  2 = Long |
| Accessibility | 1 = Difficult  2 = Easy |
| Possibility of obtaining additional benefits | 1 = Impossible  2 = Possible |
| Soil fertility retention time | 1 = Temporary (1 production campaign)  2 = Long (more than one campaign) |
| Regular control (frequency of maintenance of the plot) | 1 = Less control  2 = Regular control |
| Purchase cost CFAF per hectare | 0 ; 70,000 ; 100,000 ; 150,000 ; 220,000 |
