# Supplementary material for: Modelling smallholder farmers’ preferences for soil fertility management technologies in Benin: A stated preference approach
Source: PLoS One. 2021 Jun 30;16(6):e0253412. doi: 10.1371/journal.pone.0253412 (PMC8244892; doi:10.1371/journal.pone.0253412)
Supplement: S3 Table — (DOCX) [file pone.0253412.s007.docx]

**Table 3. Variables used in econometric models**

| **Variable** | **Modality** |
| --- | --- |
| *Status* *quo* | 1 = yes and 0 = otherwise |
| **Attributes** |  |
| Cost | Continuous variable |
| High restoration speed | 0 = Slow; 1 = Quick |
| Accessibility | 0 = Difficult; 1 = Easy |
| Possibility of obtaining additional benefits | 1 = yes and 0 = otherwise |
| Long conservation life | 0 = Temporary (one production campaign); 1 = Long (more than one campaign) |
| Maintenance frequency (regular) | 0 = Less control; 1 = Regular control |
| Maintenance frequency (regular) × Cost |  |
| Accessibility × Cost |  |
| **Physicochemical characteristics of the soil** | |
| Organic matter rate (MO) | Continuous variable |
| N | Continuous variable |
| P | Continuous variable |
| K | Continuous variable |
| Soil pH level | Continuous variable |
| Fertility level | Continuous variable (0 = weak; 1 = average; 2 = high) |
| **Socio-economic and demographic characteristics** | |
| Gender | 0 = Woman; 1 = Man; |
| Formal education | 1 = yes and 0 = otherwise |
| Number of active agricultural members | Continuous variable |
| Access to credit | 1 = yes and 0 = otherwise |
| Acreage | Continuous variable |
| Duration of fallow period | Continuous variable |
|  |  |
