# Supplementary material for: Modelling smallholder farmers’ preferences for soil fertility management technologies in Benin: A stated preference approach
Source: PLoS One. 2021 Jun 30;16(6):e0253412. doi: 10.1371/journal.pone.0253412 (PMC8244892; doi:10.1371/journal.pone.0253412)
Supplement: S4 Table — (DOCX) [file pone.0253412.s008.docx]

**Table 4. Socio-economic and demographic characteristics**

| ADH | 2 | 3 | 4 | 5 | 6 | All ADH | Statistic test |
| --- | --- | --- | --- | --- | --- | --- | --- |
| Gender (1 = man; 0 = woman) (%) | 52.19 | 50.91 | 51.67 | 51.12 | 51.79 | 51.61 | 67.36 *** |
| Class level reached (in years) | 4.67  (3.44) | 5.00 (2.44) | 6.15 (2.56) | 5.76 (3.00) | 5.96 (1.82) | 6.18 (2.80) | -3.53 *** |
| Number of active agricultural members | 12.03 (9.14) | 11.43 (5.75) | 7.56 (4.44) | 8.41 (5.49) | 8.10 (4.66) | 6.74 (5.69) | 2.78 *** |
| Access to credit (%) | 13.14 | 25.45 | 13.68 | 18.83 | 5.17 | 13.81 | 13.82 ** |
| Acreage (in Hectare) | 6.55 (4.96) | 8.81 (6.27) | 2.96 (1.55) | 1.83 (1.28) | 2.33 (1.03) | 3.8  (1.53) | 23.15 *** |

ADH = agricultural development hub
