# Supplementary material for: Modelling smallholder farmers’ preferences for soil fertility management technologies in Benin: A stated preference approach
Source: PLoS One. 2021 Jun 30;16(6):e0253412. doi: 10.1371/journal.pone.0253412 (PMC8244892; doi:10.1371/journal.pone.0253412)
Supplement: S5 Table — (DOCX) [file pone.0253412.s009.docx]

**Table 5. Calculation of Akaike (AIC), Bayesian (BIC), and Consistent (CAIC) information criteria**

| **Number of segments** | **Likelihood log** | **AIC** | **BIC** | **CAIC** |
| --- | --- | --- | --- | --- |
| 2 | −4658.60 | 9347.21 | 9398.16 | 9362.79 |
| 3 | −4607.61 | 9421.52 | 9165.34* | 9401.79 |
| 4 | −4551.67 | 9436.52 | 9318.90 | 9155.17* |
| 5 | −4545.79 | 9261.23* | 9349.90 | 9387.99 |
| 6 | −4530.58 | 9375.16 | 9169.59 | 9434.99 |

* indicates the lowest values of AIC, BIC, and CAIC
