# Supplementary material for: Modelling smallholder farmers’ preferences for soil fertility management technologies in Benin: A stated preference approach
Source: PLoS One. 2021 Jun 30;16(6):e0253412. doi: 10.1371/journal.pone.0253412 (PMC8244892; doi:10.1371/journal.pone.0253412)
Supplement: S6 Table — (DOCX) [file pone.0253412.s010.docx]

**Table 6. Estimation of the model 3 latent classes**

| **Attribute** | | Segment 1 | Segment 2 | Segment 3 |
| --- | --- | --- | --- | --- |
| Status quo | | −0.14 (0.08) ** | −3.10 (0.29) *** | −3.44 (0.32) *** |
| Cost | | 0.04 (0.02) ** | −0.05 (0.01) ** | −0.34 (0.08) *** |
| Short restoration time | | 1.17 (0.07) *** | 1.89 (0.12) *** | 3.83 (0.49) *** |
| Accessibility | | 0.33 (0.06) *** | 0.65 (0.10) *** | 0.98 (0.10) *** |
| Possibility of obtaining additional benefits | | 0.67 (0.12) *** | 0.99 (0.22) *** | 3.81 (0.49) *** |
| Long conservation time | | 0.14 (0.09) | −1.68 (0.13) *** | 0.81 (0.17) *** |
| Maintenance frequency (regular) | | 0.10 (0.07) * | 0.03 (0.01) ** | −0.11 (0.30) |
| Maintenance frequency (regular) × Cost | | 0.28 (0.35) | −1.51 (0.98) *** | −0.82 (0.01) ** |
| Accessibility × Cost | | 1.65 (1.19) ** | −0.14 (0.05) | −4.81 (2.66) ** |
| **Physicochemical characteristics of the soil** | | |  |  |
| Organic Matter (OM) Rate | −0.14 (0.24) | | 1.18 (0.40) *** |  |
| N rate | 12.88 (4.31) *** | | −1.85 (6.06) |  |
| P rate | 0.01 (0.01)* | | −0.64 (0.01)* |  |
| K rate | −1.73 (0.03) ** | | −0.19 (0.05)* |  |
| Soil pH level | 0.25 (0.09) | | −0.55 (0.12) |  |
| Fertility level | −0.46 (0.01) ** | | −0.28 (0.06) * |  |
| Duration Fallow period | −0.05 (0.15) | | −0.36 (0.08) ** |  |
| **Individual characteristics** | | |  |  |
| Gender (1 = man ; 0 = woman) | 0.42 (0.30) | | 0.62 (0.05) ** |  |
| Formal education | −1.58 (0.35) | | −0.53 (0.35) |  |
| Number of active agricultural members | 0.67 (0.18) *** | | 0.84 (0.26) *** |  |
| Access to credit | 1.42 (0.36) *** | | −0.18 (0.06) * |  |
| Acreage | 0.15 (0.19) | | 1.03 (0.20) *** |  |
| ADH3 | −0.57 (1.08)* | | 1.27 (0.83) |  |
| ADH4 | −17.40 (27.38) | | 6.79 (1.67) *** |  |
| ADH5 | 2.13 (0.69) *** | | 0.74 (0.84) * |  |
| ADH6 | 19.19 (1.40) | | 10.17 (8.68) *** |  |
| Constant | −2.21 (0.71) *** | | 0.73 (0.11) *** |  |
| **Probability of belonging to each class** | **0.33** | | **0.31** | **0.35** |
| **Number of individuals (%)** | **32.09** | | **30.95** | **36.96** |
| Number of observations | 20,940 =1047*5*4 | | | |
| Number of respondents = 962; Number of plots = 1047 | | | | |
| Likelihood log | −4322.48 | | | |
| R2 | 0.69 | | | |
| Test Wald Chi2(60) | 13587.93*** | | | |

***, **,* mean, respectively, that the coefficients are significant at the 1%, 5%, and 10% threshold; numbers in parentheses represent standard errors
