# Supplementary material for: Modelling smallholder farmers’ preferences for soil fertility management technologies in Benin: A stated preference approach
Source: PLoS One. 2021 Jun 30;16(6):e0253412. doi: 10.1371/journal.pone.0253412 (PMC8244892; doi:10.1371/journal.pone.0253412)
Supplement: S7 Table — (DOCX) [file pone.0253412.s011.docx]

**Table 7. Results of Endogenous Attribute Attendance model**

| **Attribute** | **Model 1** | | **Model 2** | |
| --- | --- | --- | --- | --- |
|  | **Coefficient** | ***p* ANA** | **Coefficient** | ***p* ANA** |
| Status quo | −3.28 (0.40) *** | 0.69 (0.03) *** | −2.74 (0.2) *** | 0.66 (0.03) *** |
| Cost | −0.10 (0.01) *** | 0.01 (0.03) | −0.09 (0.01) *** |  |
| Restoration time | 2.13 (0.09) *** | 0.07 (0.01) *** | 2.11 (0.10) *** | 0.08 (0.02) *** |
| Accessibility | 0.66 (0.13) *** | 0.10 (0.16) | 0.56 (0.03) *** | 0.02 (0.01) |
| Possibility of obtaining additional benefits | 1.92 (0.20) *** | 0.13 (0.05) ** | 1.77 (0.21) *** | 0.13 (0.05) ** |
| Soil fertility retention time | 2.95 (0.32) *** | 0.86 (0.02) *** | 1.19 (0.04) *** |  |
| Frequency of maintenance of the plot | −0.15 (0.06) ** | 0.52 (0.13) ** | −0.086 (0.05) |  |
| *p* excluded attribute |  |  |  | 0.57 (0.10) *** |
| Number of observations | 20940 |  | 20940 |  |
| Likelihood log | −4675.32 |  | −4710.56 |  |
| Wald Chi2(8) | 806.48 *** |  | 1109.68*** |  |
| AIC | 9376.65 |  | 9445.13 |  |
| BIC | 9479.99 |  | 9540.52 |  |

***, **,* mean, respectively, that the coefficients are significant at the 1%, 5% and 10% threshold; *p*: probability of ANA
