# Supplementary material for: Modelling smallholder farmers’ preferences for soil fertility management technologies in Benin: A stated preference approach
Source: PLoS One. 2021 Jun 30;16(6):e0253412. doi: 10.1371/journal.pone.0253412 (PMC8244892; doi:10.1371/journal.pone.0253412)
Supplement: S1 Appendix — (DOCX) [file pone.0253412.s012.docx]

**Appendix**

**Table A1. Average nutrient content of soils, and the degree of intensity of associated limitations, according to ADHs**

|  | ADH2  (n=279) | | ADH3  (n=56) | | ADH4  (n=345) | | ADH5  (n=254) | | ADH6  (n=113) | | **Statistic test** |
| --- | --- | --- | --- | --- | --- | --- | --- | --- | --- | --- | --- |
|  | Mean | *DIL* | Mean | *DIL* | Mean | *DIL* | Mean | *DIL* | Mean | *DIL* |  |
| **Organic matter** | 0,77 (0,53) | **IV** | 1,28 (0,68) | **III** | 1,31 (0,53) | **III** | 1,46 (0,69) | **III** | 1,32 (0,49) | **III** | 55,74 *** |
| **Nitrogen (N)** | 0,11 (0,03) | I | 0,13 (0,03) | I | 0,08 (0,03) | II | 0,08 (0,03) | II | 0,09 (0,04) | I | 65,66 *** |
| **Phosphorus (P) ass (ppm)** | 9,97 (20,40) | **IV** | 29,57 (5,8) | I | 9,64 (17,19) | **IV** | 6,44 (7,73) | **IV** | 8,40 (7,69) | **IV** | 15,76 *** |
| **Potassium (K) sample meq / 100g** | 0,22 (0,15) | **III** | 0,38 (0,32) | II | 0,23 (0,25) | **III** | 0,22 (0,17) | **III** | 0,16 (0,19) | **IV** | 10,77 *** |
| **Sum of cations meq / 100g** | 4,04 (3,90) | **IV** | 5,46 (6,17) | **III** | 4,28 (3,92) | **IV** | 7,95 (10,29) | II | 3,71 (3,23) | **IV** | 18,61 *** |
| **Saturation rate** | 52,55 (17,37) | II | 43,93 (14,27) | **III** | 70,33 (60,99) | I | 67,67 (25,40) | I | 62,85 (14,91) | I | 12,49 *** |
| **Cation exchange capacity (CEC)** | 6,48 (2,13) | **IV** | 7,85 (2,44) | **IV** | 5,91 (2,07) | **IV** | 10,06 (10,29) | **III** | 5,59 (2,63) | **IV** | 26,47 *** |
| **pH** | 6,50 (0,75) | I | 6,24 (0,63) | I | 6,30 (0,45) | I | 6,08 (0,43) | I | 6,24 (0,43) | I | 15,58 ** |

Note: (,,): standard deviation; n = number of plots; DIL: degree of intensity of limitations; Degree I (Without limitations);

Degree II (Weak limitations); Degree III (Average limitations); Degree IV (Severe Limitations);

Degree IV (Very severe limitations)
