# Supplementary material for: Modelling smallholder farmers’ preferences for soil fertility management technologies in Benin: A stated preference approach
Source: PLoS One. 2021 Jun 30;16(6):e0253412. doi: 10.1371/journal.pone.0253412 (PMC8244892; doi:10.1371/journal.pone.0253412)
Supplement: S1 File — (DOCX) [file pone.0253412.s013.docx]

**Survey tool**

**Name of investigator....................................... Date of investigation____/_____/** . **File no.Plug**

You have been selected from this community to be a respondent. The information we collect will be used for planning purposes only and your participation involves no risks or personal benefits. Responses will be treated anonymously, in the strictest confidentiality. I will be grateful if you giveprovide honest answers.

**1. CHARACTERISTICS OF THE SURVEY AREA**

| **Characteristics** | **Modalities** | **Enter the answer** |
| --- | --- | --- |
| Agricultural development pole **(PDA) ^1^** | 1=PDA1; 2=PDA2; 3=PDA3; 4=PDA4; 5=PDA5; 6=PDA6; 7=PDA7 |  |
| Department (**DEPART**) | 1=Plateau 2= Couffo 3=Mono 4=Zou 5=Colline 6=Atacora 7=Donga, 9= Alibori |  |
| Municipalities (**COMM**) | 1= Glazoué 2= Ouèssè 3= Banikoara 4= Kandi 5= Djougou 6= Kerou 7= Agbangnizoun 8= Kétou 9= Klouékanmè ; 10=Savè, 11= Ségbana; 12=Toucountouna, 13=Lalo |  |
| District (**ARRON**) | Enter the name |  |
| Village (**VILL**) | Enter the answer |  |
| Population density (data to be collected from delegates/town halls/SCDA, etc.) | Number of individuals or inhabitants in the village |  |
|  | Surface of the village (in Km²) |  |
| Access road to the village (**VOIAC**) | 1 = asphalt road, 2 = drivable track, 3 = non-drivable track, 4 = footpath, 5 = fluvial |  |
| Accessibility of the village in all seasons (**ACECC**) | 1= road in good condition and accessible in all seasons; 2= road in good condition but not accessible in all seasons; 3= road in poor condition but accessible in all seasons; 4= road in bad condition and not accessible all the year |  |
| Distance from the village to the main town (**DISTVILL**) | Enter the distance in km (**DISTVILKM**) |  |
|  | 1= ≤10 km ; 2= 10-25 km ; 3=25-30 km ; 4= >30 km. (**DISTVIL1030**) |  |
| Distance from the village to the nearest periodic market | Enter the distance in km (**DIVIMARKM**) |  |
|  | 1= ≤10 km ; 2= 10-25 km ; 3=25-30 km ; 4= >30 km. (**DIVIMAR1030**) |  |
| Availability of cultivable land in the village (**DITERV**) | 1= abundant land, 2= rare land, 3=very rare land |  |
| Existence of a grazing area in the village. (**ZONPATU**) | 0=No; 1=Yes |  |

Development Pole (PDA) Communities

**PDA 1**: Malanville and Karimama

**PDA 2** : Kandi, Banikoara, Ségbana, Gogounou, Kouandé, Kèrou Péhunco, Sinendé, Kalalé, Bembéréké

**PDA 3**: Tanguiéta- Matéri; Cobly, Boukombé; Toucountouna; Natitingou.

**PDA 4**: Tchaourou, Parakou, N'Dali, Nikki, Pèrèrè, Djidja, Savalou, Bantè, Dassa-Zoumè, Glazoué, Savè, Ouèssè, Djougou, Ouaké, Bassila and Copargo.

**PDA 5:** Covè, Zangnanando, Ouinhi, Za-Kpota, Zogbodomey, Bohicon, Abomey, Agbangnizoun, Dogbo, Aplahoué, Lalo, Klouékanmè and Djakotomey

**PDA 6**: Pobé, Kétou, Sakété, Adja-Ouèrè and Ifangni.

**PDA 7**: Avrankou, Adjarra, Akpro-Missérété, Porto-Novo, Sèmè-Podji, Aguégués, Adjohoun, Bonou, Dangbo, Zè, Allada, Toffo, Abomey-Calavi, So-Ava, Kpomassè, Tori-Bossito, Ouidah, Cotonou, Athiémé, Bopa, Comè, Lokossa, Grand-Popo, Houéyogbé and Toviklin.

**2. SOCIO-ECONOMIC CHARACTERISTICS OF THE RESPONDENT**

| **Headings** | | **Code** | | **Answer** | | | |  |
| --- | --- | --- | --- | --- | --- | --- | --- | --- |
| Name and surname of producer (**NENQ**) | | Enter the name and surname | |  | | | |  |
| Telephone number (**NPHONE**) | | Enter the answer | |  | | | |  |
| Age (**AGE**) | | Enter the age in years | | ………...Years | | | |  |
| Sex (**SEX**) | | 0=Female, 1=Male | |  | | | |  |
| Ethnicity (**ETHNIE**) | | Enter the answer | |  | | | |  |
| Marital status (**SIMATR**) | | 1=Single; 2=Married; 3=Widowed | |  | | | |  |
| Do you have access to information (radio, television, etc.)? | | 1=Yes; 0=No | |  | | | |  |
| Education level of the CM | Formal education? **(EDUCFORM)** | 1=Yes; 0=No | |  | | | |  |
|  |  | Enter the class reached (number of years) | |  | | | |  |
|  | Literate ? (**ALPHAB**) | 1=Yes; 0=No | |  | | | |  |
|  |  | If yes, what can you do? 1=read, 2=write, 3=read and write | |  | | | |  |
| Main activity (**ACTVPRCPL**) | | 1=Farmer; 2=Livestock farmer / Breeder; 3= Craftsmen; 4=Traders; 5=Other (please specify) | |  | | | |  |
| Estimate the average annual cash income from **farming** over the past 5 years | | Enter the value in FCFA (**REVVAL**) | |  | | | |  |
|  |  | Enter the corresponding code (**REVI**) 1= less than 100000; 2=100000-200000; 3=200000- 300000 ; 4=over 300000 | |  | | | |  |
|  |  | Usual part out of 10 of your agricultural income over the past 5 years (REVPAR) | |  | | | |  |
|  | | |  | **Masculin** | | | **Féminin** | |
| Number of people you **currently** feed in your consumption unit **including the head of household by age (NPERCH)** | | **Enter the number**  (**NBRP08**) 0-9 years old | |  | | |  | |
|  |  | (**NBRP914**)  9-14 years old | |  | | |  | |
|  |  | (**NBRP1460**)  14-60 years old | |  | | |  | |
|  |  | (**NBRSUP60**)  Over 60 years old | |  | | |  | |
| \| Number of **currently** active household members working permanently with you **including the head of household if he/she contributes (FTRAV)** \| \| --- \| \|  \| | | (**NBRACT914**)  9-14 years old | |  | | |  | |
|  |  | (**NBRACT1460**)  14-60 years old | |  | | |  | |
|  |  | (**NBRACSUP60**)  Over 60 years old | |  | | |  | |
| Number of years of experience in agriculture? (**NANEXP**) | | Enter the answer in year | |  | | | |  |
| Number of years of experience in soil fertility management **(NEXPFER)** | | Enter the number of years | |  | | | |  |
| Do you breed?? (**PRTQELVG**) | | 0=No; 1=Yes | |  | | | |  |
| If**YES**, name the species most commonly raised on the farm ? (**ESPELV**) | | 1=Cattle; 2=Goats; 3=Sheep; 5=Poultry; 6=Pigs; 7=Fish; 8=Other (please specify) | | **1^st^** | |  | |  |
|  |  |  |  | **2^nd^** | |  | |  |
|  |  |  |  | **3^rd^** | |  | |  |
| Possession of agricultural machinery (tractors, etc.)? (**POSMCHN**) | | 0=No; 1=Yes | |  | | | |  |
| Name three main speculations produced (in terms of income generated).  **HIERARCHIZE** (**SPECPROREV)**) | | | **Prioritize in order of importance**  1=Maize /___/ ; 2=Cassava /___/ ; 3=Soybeans /___/ ; 4=Cowpea /___/ ; 5=Rice /___/ ; 6=RaspberryPeanut /___/ ; 7=Pigeon peas ; 8=DOI (Soil Lentil) /___/ ; 9= Yam | **1^st^** |  | | |  |
|  |  |  |  | **2^nd^** |  | | |  |
|  |  |  |  | **3^rd^** |  | | |  |
| Name three main crops produced (in terms of food self-sufficiency).. **HIERARCHIZE** (**SPECPROAUTO)**) | | | **Prioritize in order of importance**  1=Maize /___/ ; 2=Cassava /___/ ; 3=Soybeans /___/ ; 4=Cowpea /___/ ; 5=Rice /___/ ; 6=RaspberryPeanut /___/ ; 7=Pigeon peas ; 8=DOI (Soil Lentil) /___/ ; 9= Yam ; 10=Sorghum /___/ ; 14=Maragrass /___/ ; 15=Corns ; 16=Other to be specified | **1^st^** |  | | |  |
|  |  |  |  | **2^nd^** |  | | |  |
|  |  |  |  | **3^rd^** |  | | |  |

**3. MEMBERSHIP IN AN ASSOCIATION AND ACCESS TO CREDIT**

| **Headings** | **Terms and conditions** | **Enter the answer** |
| --- | --- | --- |
| Are you a member of a producer group or cooperative or association? (**MBGROUP**) | 0=No; 1=Yes |  |
|  | If yes, enter the name of the group |  |
| Name the three main activities carried out by this group or association or cooperative? (**ACTGRPMT)** | **1^st^** |  |
|  | **2^nd^** |  |
|  | **3^rd^** |  |
| Have you received farm credit at least once in the last 5 years ? (**ACRED**) | 0= no; 1= yes |  |
| **If yes,** specify the average amount received by type of credit in CFA? | Formal credit (**MCREDF**) |  |
|  | Informal credit (**MCREDINF**) |  |
| Average proportion of credit allocated to soil fertility management (Share out of 10) **(PARTCREDSTRU)** | Formal credit (**PCRFORMDFER)** | ……… on 10 |
|  | Informal credit (**PCRINFFER)** | ……… on 10 |

1. **4. CONTACTS WITH INSTITUTIONS PROMOTING INNOVATIONS IN SOIL FERTILITY MANAGEMENT AND PEERS**

| **Headings** | **Terms and conditions** | **Enter the answer** | |
| --- | --- | --- | --- |
| Have you had any contacts with SCDAs, research structures, NGOs or other agricultural projects working in the field of soil fertility in the last 5 years? (**CONTSTRUC**) | 0=No; 1=Yes |  | |
| **If YES,** specify the structure(s) with which you have contact (the respondent can name several structures). **(STRUCCON**). | 1=IINRAB ; 2=SCDA ; 3=Prosol (GIZ) ; 4=Projects ; 5=NGOs; 6=Others to be specified |  | |
| Average number of visits received from these facilities per year (**NBRVSIT**) | Enter the number |  | |
| Have you participated in any training sessions in the field of soil fertility in the last 5 years? (**PARTCFORM**) | 0=No; 1=Yes |  | |
| If yes, please specify the themes on which these training sessions focused the most? (**THEMFORM**). | 1=Soil fertility management technology; 2=Technical support and advice; 3=Financial and material support; 4=Other to be specified | 1^st^ |  |
|  |  | 2^nd^ |  |
|  |  | 3^rd^ |  |
| Over the past 5 years, have you hosted experimental plots on soil fertility technologies?? (**EXPTECH**) | 0=No; 1=Yes |  |  |
| If yes, specify the technologies on which on which the experiment focuse?  (**THECEXP**). | 1=Mineral fertilizers; 2=Potassium fertilizers; 3=Biological fertilizers; 4=Microorganisms; 5=Crop rotation; 6=Use of animal manure; 7=Use of decomposed household waste; 8=Use of crop residues; 9=Composting; 10=Soil regeneration with herbaceous legumes (Mucuna, Ashynomenae, Stylosanthes etc.); 11=Soil regeneration with Pigeon pea (Cajanus Cajan); 12=Seed legumes (Acacia, Moringa, Glyricidia, Anteria etc.); 13=Spreading of decomposed cotton seeds; 14=Cassava fallow; 15=Other (specify); 16=None | 1^st^ |  |
|  |  | 2^nd^ |  |
|  |  | 3^rd^ |  |
| Do you have any contact with peer producers who have advised you on soil fertility management technologies? (**CONPAIR**). | 1=Yes 0=No |  | |
| If **YES**; indicate the technologies they advised you to use ? (**CONSEIPAIR).** | 1=Mineral fertilizers; 2=Potassium fertilizers; 3=Biological fertilizers; 4=Microorganisms; 5=Crop rotation; 6=Use of animal manure; 7=Use of decomposed household waste; 8=Use of crop residues; 9=Composting; 10=Soil regeneration with herbaceous legumes (Mucuna, Ashynomenae, Stylosanthes etc.); 11=Soil regeneration with Pigeon pea (Cajanus Cajan); 12=Seed legumes (Acacia, Moringa, Glyricidia, Anteria etc.) 12=Shrub legumes (Acacia, Moringa, glyricidia, Anterololium etc.); 13=Spreading of decomposed cotton seeds; 14=Cassava fallow; 15=Other (specify); 16=None | 1^st^ |  |
|  |  | 2^nd^ |  |
|  |  | 3^rd^ |  |

**5. SOIL FERTILITY PROBLEM**

| Name the 5 main constraints that hinder the development of your operation (rank in order of importance) (**PRINCCONTR).** | 1=Decrease in soil fertility; 2=Climatic disturbance 3=Plant diseases; 4=Use of rudimentary machines 5=Limited access to campaign credit; 6=Proliferation and persistence of weeds; 7=Destruction of crops by transhumants; 8=Lack of and high cost of labor; 9=Non-existence of a market; 10=None; 11=Other to be specified | 1^st^ |  |
| --- | --- | --- | --- |
|  |  | 2^nd^ |  |
|  |  | 3^rd^ |  |
|  |  | 4^th^ |  |
|  |  | 5^th^ |  |
| Since when the respondent been confronted with the problem of soil fertility (*if this is one of the three constraints to the development of his/her farm*)? (**PRIODINF).** | \| Enter the number of years \| \| --- \| \|  \| |  | |
| How did you notice the decrease in soil fertility on your plots? (**RMRQFER**). | 1=Decrease in yield; 2=2 = Presence of adventitia;; 3=Change in soil color; 4=Change in plant leaf color; 5=None; 6=Other to be specified | 1^st^ |  |
|  |  | 2^nd^ |  |
|  |  | 3^rd^ |  |
| Causes of declining soil fertility (**CAUZ**). | 1 = Demographic pressure;; 2=Soil erosion; 3=Misuse of fertilizers/pesticides; 4=Deforestation; 5=Overexploitation of land; 6=Expensive or difficult access to organic fertilizers (animal dung, crop residues, etc.); 7=None; 8=Other to specify | 1^st^ |  |
|  |  | 2^nd^ |  |
|  |  | 3^rd^ |  |

**6. CHARACTERISTICS OF THE SURVEYE'S PLOTS 2(At this level, all the respondent's plots are to be taken into account)**

**6.1. PHYSICAL AND BIOLOGICAL CHARACTERISTICS**

***NB: The field is different from the plot: several parcels can be found in one field; Continuous numbering when passing from one plot to another***

| \| Field \| \| --- \| \| \| number \| \| | \| Plot \| \| --- \| \| \| number \| \| | \| Method of acquisition of the plot \| \| --- \| \| \|  \| \| Enter the answer \|   **(1)** | Area in Ha | Level of Soil fertility? **(2)** | Types of labor more used for soil fertilization operations**?**  **(3)** | | | Propriété physique du sol | | | | | | | | | Method used to protect the soil against erosion?  Enter the answer **(12)** |
| --- | --- | --- | --- | --- | --- | --- | --- | --- | --- | --- | --- | --- | --- | --- | --- | --- | --- | --- | --- | --- | --- | --- | --- | --- |
|  |  |  |  |  |  |  |  | Ecology **(4)** | Type of **land(5)** | Type of soil **(6)** | Soil texture **(7)** | Color **(8)** | Porosity level  Enter the answer  **(9)** | Presence of organic matter (residues, old roots, straw)  Enter the answer (*1=Yes, 0=No*) | If organic matter present, specify the state  Enter the answer **(10)** | Niveau de Présence de vers de terre/ Micro-organismes  Enter the answer **(11)** |  |
|  |  |  |  |  |  |  |  |  |  |  |  |  |  |  |  |  |  |
|  |  |  |  |  | 1^er^ | 2^ième^ | 3^ième^ |  |  |  |  |  |  |  |  |  |  |
|  |  |  |  |  |  |  |  |  |  |  |  |  |  |  |  |  |  |
|  |  |  |  |  |  |  |  |  |  |  |  |  |  |  |  |  |  |
|  |  |  |  |  |  |  |  |  |  |  |  |  |  |  |  |  |  |
|  |  |  |  |  |  |  |  |  |  |  |  |  |  |  |  |  |  |
|  |  |  |  |  |  |  |  |  |  |  |  |  |  |  |  |  |  |
|  |  |  |  |  |  |  |  |  |  |  |  |  |  |  |  |  |  |

1. ***Mode of acquisition****: (1=purchase, 2=inheritance, 3=donation (definitive transfer), 4=rental (temporary transfer with consideration), 5= loan, 6=sharecropping (Specify rent paid* *and % of crop), 7=pledge, 8= free loan or without consideration; 9=Other (Specify)*
2. ***Soil fertility level*** *(1=Very fertile, 2=* *2 = Not very fertile,, 3=Infertile)*
3. ***Types of labor most used for soil fertilization operations*** *(1 = occasional employee,, 2=Family, 3=Mutual, 4=None; 5=Other)*
4. ***Ecology****: 1=Plain, 2 = Pluvial assisted irrigation, 3 = Non-irrigated lowlands, 4 = Irrigated lowlands, 5 = Mangrove, 6 = Other (specify)*
5. ***Type of land*** *(1=Flat; 2=Low slope; 3=High slope)*
6. ***Soil type****:(1=Tropical Ferruginous soils; 2=Little developed soils; 3=Ferralitic soils; 4=Hydromorphic soils; 5=Vertisols)*
7. ***T exture****: (1=Slit; 2=Clay; 3=Sand)*
8. ***Color*** *(1=Dark; 2=Light; 3=Red; 4=Other to specify)*
9. ***Porosity level****? (1=Non-porous soil; 2=Low; 3=Medium; 4=High)*
10. ***State of organic matter on the soil*** *(1=Fresh, 2=Dry; 3=Little decomposed, 4=Decomposed)*
11. ***Level of presence of earthworms/microorganisms (****1=Absent; 2=Slightly present; 3=Abundant)*
12. ***Method of protection*** *(1=Vegetation cover, 2=Residue left on surface, 3=Stone strings; 4=None; 5=Other to be specified)*

**All sub-questions in Question 6 are for all of the respondent's plots except those related to soil testing. Soil sampling will only be done on sampled plots whose soils will be analyzed.**

**PHYSICAL CHARACTERISTICS (CONTINUED AND END)**

| Field Number | Plot Number | Crop system **(1)** | Crops grown on the plot **(2)** | | Main production objective? **(3)** | Do you use improved seeds?  Enter the answer (*1=Yes, 0=No*) | Plant leaf color **(4)** | Have you practiced crop rotation on this plot in the last 3 years in order to regenerate soil fertility? | | | | Compost or manure inputs in the last 3 years?  Enter the answer (*1=Yes, 0=No*) | Have you practiced fallowing in the last 3 years? | | | Crop residue inputs in the last 3 years?  Enter the answer (*1=Yes, 0=No*) | Intakes of? household waste over the past 3 years in the last 3 years ?  Enter the answer (*1=Yes, 0=No*) | Soil regeneration with herbaceous legumes (Mucuna, Ashynomenae, Stylosanthes etc.) in the last 3 years?  Enter the answer (*1=Yes, 0=No*) | Cultivation of corridor/fence woody legumes (Acacia, Moringa, glyricidia, Anterololium etc.) in the last 3 years on this plot ?  Enter the answer (*1=Yes, 0=No*) |
| --- | --- | --- | --- | --- | --- | --- | --- | --- | --- | --- | --- | --- | --- | --- | --- | --- | --- | --- | --- |
|  |  |  | Cult1 | Cult  2 |  |  |  |  |  |  |  |  |  |  |  |  |  |  |  |
|  |  |  |  |  |  |  |  |  |  |  |  |  | Enter the answer (*1=Yes, 0=No*) | If **yes**, specify type of fallow?  **(6)** | If **yes**, specify the duration(mois) ? |  |  |  |  |
|  |  |  |  |  |  |  |  | Enter the answer (*1=Yes, 0=No*) | If yes, most used crops?  **(5)** | | |  |  |  |  |  |  |  |  |
|  |  |  |  |  |  |  |  |  | 1st | 2nd | 3rd |  |  |  |  |  |  |  |  |
|  |  |  | : | : |  |  |  |  |  |  |  |  |  |  |  |  |  |  |  |
|  |  |  |  |  |  |  |  |  |  |  |  |  |  |  |  |  |  |  |  |
|  |  |  |  |  |  |  |  |  |  |  |  |  |  |  |  |  |  |  |  |
|  |  |  |  |  |  |  |  |  |  |  |  |  |  |  |  |  |  |  |  |
|  |  |  |  |  |  |  |  |  |  |  |  |  |  |  |  |  |  |  |  |
|  |  |  |  |  |  |  |  |  |  |  |  |  |  |  |  |  |  |  |  |
|  |  |  |  |  |  |  |  |  |  |  |  |  |  |  |  |  |  |  |  |

1. ***Cropping system****: 1=Pure crop; 2=Associated crops; 3=Crop rotation; 4=Fallow; 5=Agro-forestry; 6=Other (please specify)*
2. ***Crop grown on the plot****: 1=Maize; 2=Cassava; 3=Cowpea; 4=Peanut; 5=Soybeans; 6=Vouandzou; 7=Pigeon peas; 8=Rice; 9=DOI; 10=No crop; 11=Other to be specified*
3. ***Production objective****. (1=sale, 2=self-consumption, 3=sale and self-consumption, 4=livestock feed; 5=other to be specified)*
4. ***Plant leaf color*** *(1= dark green, 2= light green, 3= yellow)*
5. ***Most commonly used crops:*** *1=Maize; 2=Cassava; 3=Cowpea; 4=Peanut; 5=Soybeans; 6=Vouandzou; 7=Pigeon peas; 8=Rice; 9=DOI; 10=None; 11=Other to be specified)*
6. ***Specify type of fallow most adopted: (****1=Natural fallow; 2=Cassava fallow; 3=Pigeon pea (Cajanus Cajan) fallow; 4=Acacia fallow; 5=Oil palm fallow. 6=Other to specify)*

**7. IMPORTANCE OF CHARACTERISTICS OF SOIL FERTILITY MANAGEMENT PRACTICES IN THE DECISION OF USE, ABANDONMENT OR NON-USE BY PRODUCERS**

Indicate the importance ("**3=important**" or "**2=not very important**", "**1=not at all important**") and rankings of the characteristics of a soil fertility management technology, contained in the table below in your decision **to use,** **abandon, or not use it**. ***(Be sure to define the criteria to producers)***

***Definition of possible selection criteria***

1. **Speed of fertility restoration**: duration of soil fertility rehabilitation
2. **Accessibility**: availability of materials used in the realization of the technology (e.g. cow dung, mucuna seed, etc.)
3. **Possibility of obtaining edible or marketable by-products:** some soil fertility management technologies facilitate the obtaining of edible or marketable products (cassava, cowpea, soybeans, firewood, fodder, etc.), contributing to increased household income and food security. On the other hand, others do not facilitate the production of edible products (e.g., mucuna).
4. Soil **fertility retention time:** effectiveness of the technology over one or more production seasons after application
5. **Regular control**: frequency of maintenance of the plot
6. **Cost of realization of** the technology per hectare (FCFA) : investment amount
7. **Level of fertility recovery**
8. **Requires the use of production factors for the realization (labor, machines, etc.)**
9. **Possibility to use crop residues or to promote the production of fodder**

| **Features** | | **Relative importance in the decision to use, abandon or not use ("3=very important", "2=not important" and "1=not important at all")** | **Rank** | **Justification** |
| --- | --- | --- | --- | --- |
| 1 | Recovery speed |  |  |  |
| 2 | Accessibility |  |  |  |
| 3 | Possibility of obtaining an edible or marketable product |  |  |  |
| 4 | Duration of the conversation |  |  |  |
| 5 | Regular control (frequency of maintenance of the plot) |  |  |  |
| 6 | Cost (FCFA/HA) |  |  |  |
| 7 | Level of fertility recovery |  |  |  |
| 8 | Requires the use of production factors for the realization (labor, machines, etc.) |  |  |  |
| 9 | Possibility to use crop residues or to promote the production of fodder |  |  |  |

**8. CHARACTERISTICS OF THE MAIN TECHNOLOGY CURRENTLY USED ON EACH PLOT (status *qu0*) (At this point, consider the number of plots to be sampled at this producer level)**

| Field Number | Plot Number | Main technology you currently use most for soil fertility management *Qu0*? **(1)** | Specify the soil fertility restoration rate of this technology ?  Enter the answer **(1=Slow; 2=Fast)** | Indicate the accessibility of the technology  Enter the answer **(1=Easy; 2=Difficult)** | Do you have the possibility of obtaining edible or marketable by- products after using this technology ?  Enter the answer **(1=Yes; 0=No)** | Specify the duration of soil fertility conservation after application of this technology?  Enter the answer  **(1=Long *one campaign*; *2=Temporary more than one campaign***) | This technology requires regular control of the field with its application (frequency of maintenance of the plot)?  Enter the answer  **(1=Less control; 2=Regular control)** | Estimate the cost of realization/purchase of this  technology in CFA per Hectare? |
| --- | --- | --- | --- | --- | --- | --- | --- | --- |
|  |  |  |  |  |  |  |  |  |
|  |  |  |  |  |  |  |  |  |
|  |  |  |  |  |  |  |  |  |

**Main technologies used***: (1=Chemical* *fertilizers; 2=Potassium fertilizers; 3=Organic fertilizers; 4=Microorganisms; 5=Crop rotation; 6=Use of animal manure; 7=Use of decomposed* *household waste; 8=Use of crop residues; 9=Use of compost; 10=Soil regeneration with herbaceous legumes (Mucuna, Ashynomenae, Stylosanthes etc.); 11=Soil regeneration with Pigeon pea (Cajanus Cajan); 12=Natural fallow land; 13=Shrub legumes (Acacia, Moringa, etc.); 14=Seed legumes (Acacia, Moringa etc.); 15=Seed legumes (Acacia, Moringa etc.).);; 16=Oil palm fallow; 17= burning practice; 18=Other (please specify)*

**9. EXPERIMENTAL CHOICE OF SOIL FERTILITY MANAGEMENT PRACTICES (The same:** **At this level, consider the number of plots to be sampled at this producer level)**

You are offered several sets of choices. For each set of choices, you are given 5 possible options (Practice **1; 2; 3; 4**; or not interested in any of the proposed practices). Each possible option is defined by a set of criteria. For each set of choices, be sure to analyze and appreciate the different practices presented in each group in order to make an accurate choice. The choice will be made for each plot selected for this study, the characteristics of which were described in Section 6. Note that the practices proposed here are for hypothetical purposes. The experiment would have no real immediate impact on your operations. The results will be used more generally to determine the appropriate practice for soil fertility management in your community.


| Choice set 1 | | | | | | |
| --- | --- | --- | --- | --- | --- | --- |
|  | | Practice **#1**  **Woody legumes (Acacia, Moringa, Gliricidia, Anterololium eg.)** | Practice **#2**  **Mineral fertilizers** | **Practice #3**  **Crop residues** | **Practice #4** | None of these practices interest me, i prefer to maintain current practice  (*Option q0)* |
| **Restoration time** | | Slow | Quick | Quick | Slow |  |
| **Accessibility** | | Easy | Difficult | Easy | Difficult |  |
| **Possibility of obtaining edible by-products** | | Yes | Yes | Yes | No |  |
| **Soil fertility**  **retention time** | | Long (more than one production campaign) | Temporary (1 production campaign) | Long (more than one production campaign) | Temporary (1 production campaign) |  |
| **Regular control (**frequency of maintenance of the plot) | | Less | Regular | Regular | Less |  |
| **Purchase cost**  **(CFAF per hectare)** | | 200 000 | 100 000 | 200 000 | 70 000 |  |
| ***Which of these***  ***practices do***  ***you choose?***  ***Justify your Choice*** | ***Plot1*** |  |  |  |  |  |
|  | ***Plot2*** |  |  |  |  |  |
|  | ***Plot3*** |  |  |  |  |  |

| Choice set 2 | | | | | | |
| --- | --- | --- | --- | --- | --- | --- |
|  | | Practice **#5** | Practice **#6 Soil regeneration**  **with herbaceous**  **legumes**  **(Mucuna,**  **Ashynomenae,**  **Stylosanthes etc.)** | **Practical #7**  **Microorganism**  **(vermiculture or**  **mushrooms, etc.)** | **Practice #8**  **Crop rotation** | None of these practices interest me, i prefer to maintain current practice  (*Option q0)* |
| **Restoration time** | | Slow | Quick | Slow | Quick |  |
| **Accessibility** | | Easy | Difficult | Difficult | Easy |  |
| **Possibility of obtaining edible by-products** | | No | No | Yes | Yes |  |
| **Soil fertility**  **retention time** | | Temporary  (1 production  campaign) | Temporary  (1 production  campaign) | Long (more than one  production campaign) | Long  (more than one  production campaign) |  |
| **Regular control (**frequency of maintenance of the plot) | | Regular | Less | Regular | Less |  |
| **Purchase cost**  **(CFAF per hectare)** | | 70 000 | 70 000 | 100 000 | 70 000 |  |
| ***Which of these***  ***practices do***  ***you choose?***  ***Justify your Choice*** | ***Plot1*** |  |  |  |  |  |
|  | ***Plot2*** |  |  |  |  |  |
|  | ***Plot3*** |  |  |  |  |  |

| Choice set 3 | | | | | | |
| --- | --- | --- | --- | --- | --- | --- |
|  | | Practice **#9** | **Practice #10**  **Organic fertilizers** | **Practical #11**  **Natural fallow** | **Practical #12**  **Use of animal manure /**  **Garbage / Compost** | None of these practices interest me, i prefer to maintain current practice  (*Option q0)* |
| **Restoration time** | | Slow | Quick | Slow | Quick |  |
| **Accessibility** | | Difficult | Easy | Easy | Difficult |  |
| **Possibility of obtaining edible by-products** | | Yes | Yes | No | Yes |  |
| **Soil fertility**  **retention time** | | Long (more than one production campaign) | Temporary (1 production  campaign) | Long (more than one production campaign) | Long (more than one production campaign) |  |
| **Regular control (**frequency of maintenance of the plot) | | Less | Regular | Less | Regular |  |
| **Purchase cost**  **(CFAF per hectare)** | | 100 000 | 100 000 | 0 | 220 000 |  |
| ***Which of these***  ***practices do***  ***you choose? Justify your Choice*** | ***Plot1*** |  |  |  |  |  |
|  | ***Plot2*** |  |  |  |  |  |
|  | ***Plot3*** |  |  |  |  |  |

| Choice set 4 | | | | | | |
| --- | --- | --- | --- | --- | --- | --- |
|  | | Practical **#13** | Practical #14 | **Practical #15**  **Potassium fertilizer** | Practical **#16** | None of these practices interest me, i prefer to maintain current practice  (*Option q0)* |
| **Restoration time** | | Quick | Slow | Quick | Slow |  |
| **Accessibility** | | Difficult | Difficult | Easy | Easy |  |
| **Possibility of obtaining edible by-products** | | Yes | No | Yes | Yes |  |
| **Soil fertility**  **retention time** | | Temporary (1 production campaign) | Long  (more than one production campaign) | Temporary (1 production campaign) | Temporary (1 production campaign) |  |
| **Regular control (**frequency of maintenance of the plot) | | Less | Regular | Less | Regular |  |
| **Purchase cost**  **(CFAF per hectare)** | | 70 000 | 70 000 | 150 000 | 150 000 |  |
| ***Which of these***  ***practices do***  ***you choose? Justify your Choice*** | ***Plot1*** |  |  |  |  |  |
|  | ***Plot2*** |  |  |  |  |  |
|  | ***Plot3*** |  |  |  |  |  |

**MEASURES TO PROMOTE THE LARGE-SCALE ADOPTION OF FERTILITY MANAGEMENT AND SOIL CONSERVATION TECHNOLOGIES**

| **Headings** | **Terms and conditions** | **Rank** | | **Justification** |
| --- | --- | --- | --- | --- |
|  |  |  |  |  |
| Technical | 1= Technical performance;  2= Relevance to producers expectation | **1st** |  |  |
|  |  |  |  |  |
| measures | 3= Frequent support from extension and technical agents;  4= Installation of |  |  |  |
|  |  | **2nd** |  |  |
|  | school plots; 5= Sharing experiences with producer pairs;  6= Others to be specified |  |  |  |
|  |  |  |  |  |
|  |  |  |  |  |
|  |  | **3rd** |  |  |
|  |  |  |  |  |
|  |  |  |  |  |
| Economic | 1=Installation cost;  2=Easier access to agricultural credit;  3=Financial support (grants, donations, etc.);  4=Presentation of technologies at an acceptable cost; | **1st** |  |  |
|  |  |  |  |  |
| measures | 5=Easier access to microfinance institutions; 6=Other to be specified |  |  |  |
|  |  | **2nd** |  |  |
|  |  |  |  |  |
|  |  | **3rd** |  |  |
|  |  |  |  |  |
|  |  |  |  |  |
| Institutional and | 1= Promote the benefits of the technologies through information | **1st** |  |  |
|  |  |  |  |  |
| policy measures | dissemination channels or awareness and outreach campaigns;  2= Facilitate access in villages; |  |  |  |
|  |  | **2nd** |  |  |
|  | 3=Other to be specified |  |  |  |
|  |  |  |  |  |
|  |  |  |  |  |
|  |  | **3rd** |  |  |
|  |  |  |  |  |

Your opinion on the experience of **comments from the respondent ………………………………………………………………………………………………………………………………………………………………………**

1. **Specify the number of plots to be sampled at this producer level?**
2. **Specify the number of the first plot?**
3. **Specify the number of the second plot?**
4. **What is the number of the third plot?**
5. **Have you taken samples from this producer's plots? (Yes or No)**

**Thank you for your availability.**
