# Supplementary material for: Modelling smallholder farmers’ preferences for soil fertility management technologies in Benin: A stated preference approach
Source: PLoS One. 2021 Jun 30;16(6):e0253412. doi: 10.1371/journal.pone.0253412 (PMC8244892; doi:10.1371/journal.pone.0253412)
Supplement: Attachment — Submitted filename: Response to Reviewers.docx [file pone.0253412.s014.docx]

**Reply to reviewers’ comments on PONE-D-21-07803R1**

**“Modelling Smallholder Farmers' Preferences for Soil Fertility Management Technologies in Benin: A Stated Preference Approach.”**

**Response to academic editor**

***Reply to specific comments:***

1. We note the following text is only included in the Tracked Changes document: « The study was carried out on private land. We confirm that, the owner of the land gave permission to conduct the study on this site, and no specific permissions were required for these locations/activities. The agents of the research centers, the town hall, the village chiefs, and the resource persons facilitated the interviews with the producers in the villages."

Please confirm and include the above permissions text in the Methods section of your manuscript.

- **Response 1:** The correction has been made as suggested. We confirm and include the following text concerning the permissions text in the Methods section of the manuscript. " The study was carried out on private land. We confirm that, the owner of the land gave permission to conduct the study on this site, and no specific permissions were required for these locations/activities. The agents of the research centers, the town hall, the village chiefs, and the resource persons facilitated the interviews with the producers in the villages."

**Reply to reviewer 1:**

First, we want to thank te reviewer for the time spent going through this paper for comments. We are convinced that these comments have substantially improved the paper.

Abstract and Introduction improvement:

I am glad to assess this informative study. In my opinion, I have some guidelines for the authors to enhance the study quality before endorsing it for publication. As the Abstract is the main door or "FACE" of the manuscript, it should briefly present high-quality English with new information. I am recommending the authors of this study to expand Abstract, as it is too short. The Abstract should be around 250 words. I have suggested some studies to check the abstracts and improve yours and cite them in the introduction and build your study objectives like these studies.

Hussain, T., Abbas, J., Wei, Z., & Nurunnabi, M. (2019). The Effect of Sustainable Urban Planning and Slum Disamenity on The Value of Neighboring Residential Property: Application of The Hedonic Pricing Model in Rent Price Appraisal. Sustainability, 11(4). doi:10.3390/su11041144
Abbas, J., Raza, S., Nurunnabi, M., Minai, M. S., & Bano, S. (2019). The Impact of Entrepreneurial Business Networks on Firms’ Performance Through a Mediating Role of Dynamic Capabilities. Sustainability, 11(11). https://doi.org/10.3390/su11113006
Hussain, T., Abbas, J., Wei, Z., Ahmad, S., Xuehao, B., & Gaoli, Z. (2021). Impact of Urban Village Disamenity on Neighboring Residential Properties: Empirical Evidence from Nanjing through Hedonic Pricing Model Appraisal. Journal of Urban Planning and Development, 147(1), 04020055. https://doi.org/10.1061/(asce)up.1943-5444.0000645

- **Response :** We thank the reviewer for this proposal. We expand the Abstract as suggested. Some studies suggested by the reviewer have been exploited and cited in order to improve the abstract and the introduction and build our study objectives.

Literature section

It presents a good summary of the literature. I suggest authors add the literature as recommended below to improve the manuscript. Overall, the authors have creatively linked variables. It reflects an innovative model of the study. I am pleased to read this article. However, I have some suggestions for the authors to enhance the quality of the literature section. The authors can add few lines about technological innovations and environmental responsibility practices. Please see the suggested studies and cite them to enhance the literature section.
Abbas, J., Zhang, Q., Hussain, I., Akram, S., Afaq, A., & Shad, M. A. (2020). Sustainable Innovation in Small Medium Enterprises: The Impact of Knowledge Management on Organizational Innovation through a Mediation Analysis by Using SEM Approach. Sustainability, 12(6). https://doi.org/10.3390/su12062407

- **Response :** The correction has been made as suggested by the reviewer. We have added the literature as recommended

The results section of the paper presents a good view of the study. This work presents a notable investigation on a selected topic. I suggest including some graphical presentations to improve the quality of this study. Please see the proposed studies and see the graphical representation. Improve your work like these studies and cite them in this section.

Abbasi, K. R., Abbas, J., & Tufail, M. (2021). Revisiting electricity consumption, price, and real GDP: A modified sectoral level analysis from Pakistan. Energy Policy, 149, 112087. doi:10.1016/j.enpol.2020.112087

Abbas, J., Aman, J., Nurunnabi, M., & Bano, S. (2019). The Impact of Social Media on Learning Behavior for Sustainable Education: Evidence of Students from Selected Universities in Pakistan. Sustainability, 11(6). https://doi.org/10.3390/su11061683

Abbasi, K. R., Hussain, K., Abbas, J., Adedoyin, F. F., Shaikh, P. A., Yousaf, H., & Muhammad, F. (2021). Analyzing the role of industrial sector's electricity consumption, prices, and GDP: A modified empirical evidence from Pakistan [J]. AIMS Energy, 9(1), 29-49. doi:10.3934/energy.2021003
Abbas, J., Mahmood, S., Ali, H., Ali Raza, M., Ali, G., Aman, J., . . . Nurunnabi, M. (2019). The Effects of Corporate Social Responsibility Practices and Environmental Factors through a Moderating Role of Social Media Marketing on Sustainable Performance of Business Firms. Sustainability, 11(12), 3434.

**Response :** The observation are very relevant and has been taken into account as suggested by the reviewer. Some graphical presentations are now added to improve the quality of this study. Some studies suggested by the reviewer have been exploited and cited in the manuscript

Conclusion
I suggest you make a separate heading of the conclusion and do not mix it with implications.

- **Response :** The correction has been made as suggested by the reviewer.

Policy Recommendations

I again recommend you to make a separate heading of the Policy Recommendations.

The conclusion section is acceptable. Overall, this presents a good piece of research work. I recommend that authors do a little more work and revise this article accordingly. I suggest the authors check English quality and fix some weak sentences. If you have already taken English editing service, ask them to recheck the quality to meet scientific merit for publication. I endorse this manuscript for publication after minor corrections, as suggested.

- **Response :** The correction is done as suggested by the reviewer. We have made a separate heading of the Policy Recommendations. We have checked English quality and fix some weak sentences with English editing service

**Response to Reviewer #2 Comments**

I am glad to review and assess this interesting article, entitled, Modeling Smallholder Farmers' Preferences for Soil Fertility Management Technologies in Benin: A Stated Preference Approach. This study analyses the most interesting technological profiles to offer to each category of producer in Benin agricultural development hubs (ADHs) using the stated preference method, more precisely, the improved choice experiment method. The organization of this article is good and satisfactory. The Introduction section and methodology portions are adequate. I suggest the authors improve the Materials and Methods section by adding some latest articles' citations to enhance the work quality and also concise this part. Also, Improve the Conclusion part as well. Overall, the manuscript is a good piece of work. I recommend that authors do a little more work and add the latest literature to support the study, as suggested. The English level is good and smooth, e.g., the language standard, specifically the grammar, of sufficient quality to meet scientific merit for publication. I accept this manuscript after minor revision, as I have recommended.

***Reply to general comments:***

- First, we express our gratitude to the reviewer for devoting his time to this paper for the comments and for providing many relevant articles to the topic addressed in this paper. We are convinced that these comments and the review of suggested articles have substantially improved the paper. The correction has been made as suggested. We have improved the Materials and Methods, and Conclusion section, by adding some latest articles' citations to enhance the work quality and also concise this part.
